# Hypoxia-induced release, nuclear translocation, and signaling activity of a DLK1 intracellular fragment in glioma

**DOI:** 10.1038/s41388-020-1273-9

**Published:** 2020-03-24

**Authors:** Elisa Stellaria Grassi, Vasiliki Pantazopoulou, Alexander Pietras

**Affiliations:** 1 Department of Laboratory Medicine, Division of Translational Cancer Research, Lund University, Lund, Sweden; 2 Present Address: Department of Clinical Sciences and Community Health (DISCCO), University of Milan, Milan, Italy

**Keywords:** CNS cancer, Cancer microenvironment

## Abstract

Glioblastoma multiforme is characterized in part by severe hypoxia associated with tumor necrosis. The cellular response to hypoxia can influence several properties of tumor cells associated with aggressive tumor growth, including metabolic adaptations and tumor cell migration and invasion. Here, we found that Delta Like Non-Canonical Notch Ligand 1 (DLK1) expression was elevated as compared with normal brain in a genetically engineered mouse model of glioma, and that *DLK1* expression increased with tumor grade in human glioma samples. DLK1 expression was highest in hypoxic and perivascular tumor areas, and we found that hypoxia induced the release and nuclear translocation of an intracellular fragment of DLK1 in murine glioma as well as in human glioma cultures. Release of the intracellular fragment was dependent on ADAM17 and Hypoxia-inducible Factor 1alpha and 2alpha (HIF-1alpha/HIF-2alpha), as ADAM17 inhibitors and *HIF1A*/*HIF2A* siRNA blocked DLK1 cleavage. Expression of a cleavable form of DLK1 amplified several hypoxia-induced traits of glioma cells such as colony formation, stem cell marker gene expression, a PI3K-pathway-mediated metabolic shift, and enhanced invasiveness. Effects of DLK1 were dependent on DLK1-cleavage by ADAM17, as expression of non-cleavable DLK1 could not replicate the DLK1-induced hypoxic phenotype. Finally, forced expression of DLK1 resulted in more invasive tumor growth in a PDGFB-induced glioma mouse model without affecting overall survival. Together, our findings suggest a previously undescribed role for DLK1 as an intracellular signaling molecule.

## Introduction

High-grade gliomas represent a devastating disease with little improvement in overall survival over the past several decades [1]. Clinical challenges associated with the treatment of glioma can be traced back to biological properties of glioma cells, like inherent therapeutic resistance and a high migratory capacity, as well as to microenvironmental realities of gliomas, such as excessive areas of hypoxia associated with necrosis, a pathognomonic feature of the highest-grade gliomas (GBM) [2]. These properties may be causally related: evidence supports an increasing role for microenvironmental factors like hypoxia in regulating oncogenic properties of brain tumor cells [3]. Despite a fundamental understanding of the cellular response to hypoxia as orchestrated by the hypoxia-inducible transcription factors (mainly HIF-1α and HIF-2α) [4], hypoxia-induced tumor aggressiveness is only partially understood.

Delta Like Non-Canonical Notch Ligand 1 (DLK1) is a transmembrane protein previously associated with glioma progression [5]. Its expression is associated with tumor cell properties like migration, invasion, and stemness in cancer [6–9], however, many outstanding questions remain regarding the function and regulation of DLK1. While many functions of DLK1 appear related to its potential regulation of Notch signaling [10], other studies have found no evidence of Notch dependency for DLK1 [11], suggesting that the role of DLK1 can vary. Signaling from DLK1 can involve ADAM17-mediated release of its extracellular domain [12, 13], with best-described consequences in inhibition of adipocyte differentiation. Mechanisms involve activation of the MEK/ERK pathway and Sox9 [14–16]. The ligand for soluble DLK1 remains unknown, but its signaling has been described to depend on DLK1 expression itself on the receiving cell [11], and DLK1 can interact with DLK1 itself [17] as well as Fibronectin [18], thereby leading to activation of integrin signaling [18]. The function of the intracellular domain (ICD) remains elusive.

Here, we used mouse models of glioma and human GBM cultures to characterize the expression, regulation, and function of DLK1 in GBM.

## Results

### DLK1 is overexpressed in GBM and its subcellular localization is hypoxia-dependent

**Fig. 1:**
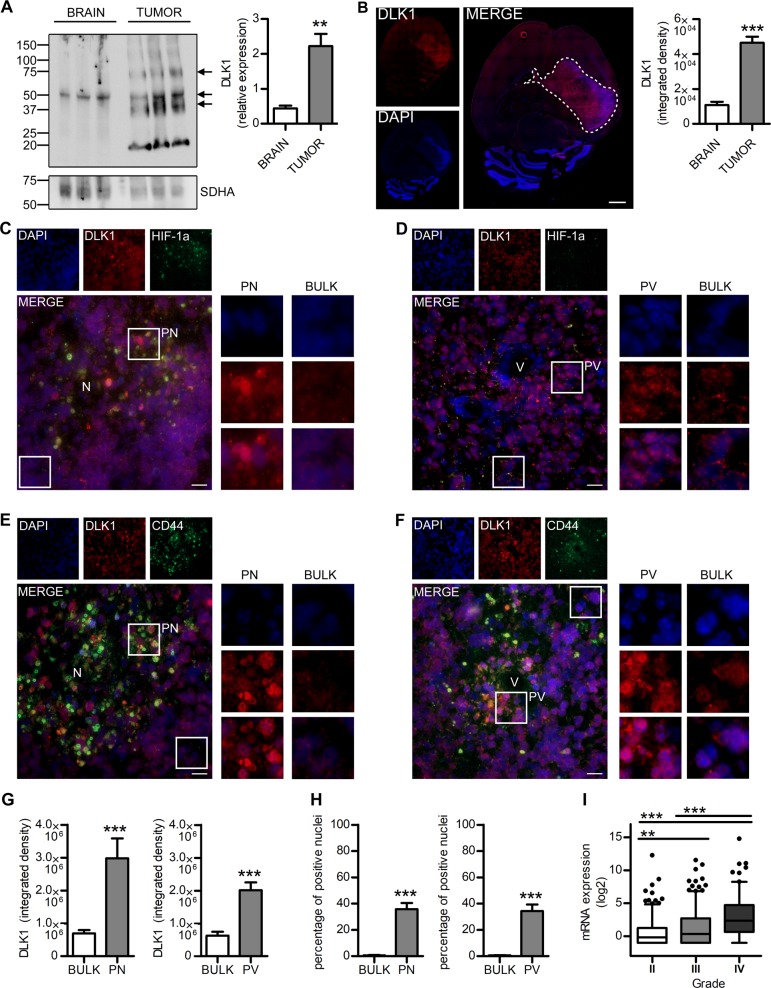
DLK1 is overexpressed in murine GBM and has nuclear localization in the perivascular and hypoxic niches. **a** Representative images and densitometric analysis of western blots showing DLK1 expression levels in healthy brains and PDGFB-induced murine gliomas. SDHA was used as loading control. **b** Representative images and signal intensity quantification of immunofluorescent stainings showing DLK1 expression levels in brains with PDGFB-induced gliomas. Dashed line encircles tumor area. Scale bars represent 1 mm. **c**–**f** Representative images showing DLK1 immunofluorescent staining in perivascular and hypoxic niches, identified by co-localization with HIF-1α (**c**, **d**) and CD44 (**d**, **e**) stainings. For each image, the high magnification inserts show DLK1 nuclear localization. V vessel, N necrosis. Scale bars represent 20 µm. **g** Signal intensity quantification of the above described immunofluorescent stainings showing DLK1 nuclear expression levels in perinecrotic, perivascular and bulk cellular tumor areas. **h** Percentage of DLK1 positive nuclei in perinecrotic, perivascular and bulk cellular tumor areas of the above described immunofluorescent stainings. **i** Boxplots showing DLK1 expression levels in 620 human gliomas from TCGA database, stratified by tumor grade. Statistical analysis: **a**, **b**, all data are from three independent experiments, **g**, **h** All data are from three independent experiments, of each three unrelated areas were analyzed. All experiments expressed as mean ± SEM, statistical significance was determined by *t*-test with Welch’s correction applied for **g**–**h**. **i** Data from 620 patients in TCGA database, grade II *n* = 226, grade III *n* = 244, grade IV *n* = 150, statistical significance was determined by Tukey’s HSD using the GlioVis tool. In the whole figure significance is represented as **p < 0.01 and ***p < 0.001.

Murine gliomas were generated using RCAS/tv-a to overexpress PDGFB and induce p53/PTEN loss in Nestin-expressing cells of Nestin-*tv-a* (*Ntv-a*) mice [19, 20]. Western blots revealed enhanced DLK1 expression in tumors compared with surrounding normal brain (Fig. 1a). The band pattern was more complex in tumor samples compared with controls, indicating tumor-specific DLK1 regulation. Immunofluorescent stainings showed enhanced DLK1 signal in tumors, with some intratumoral heterogeneity (Fig. 1b). Co-staining against HIF-1α, a hypoxia marker, and CD44, a marker previously used to define perivascular and perinecrotic tumor areas [20], revealed that DLK1 localized prevalently in perivascular and hypoxic niches, with previously unreported nuclear localization specifically in these areas (Fig. 1c–h). Analysis of DLK1 expression in the Cancer Genome Atlas (TCGA [21]), in the Chinese Glioma Genome Atlas (CGGA [22]) datasets and evaluation of DLK1 stainings in low- vs high-grade murine tumors revealed higher DLK1 expression in high-grade gliomas, that typically have hypoxic areas (Fig. 1i and Supplementary Fig. 1).

### Hypoxia-induced release and nuclear translocation of a DLK1 intracellular fragment

**Fig. 2:**
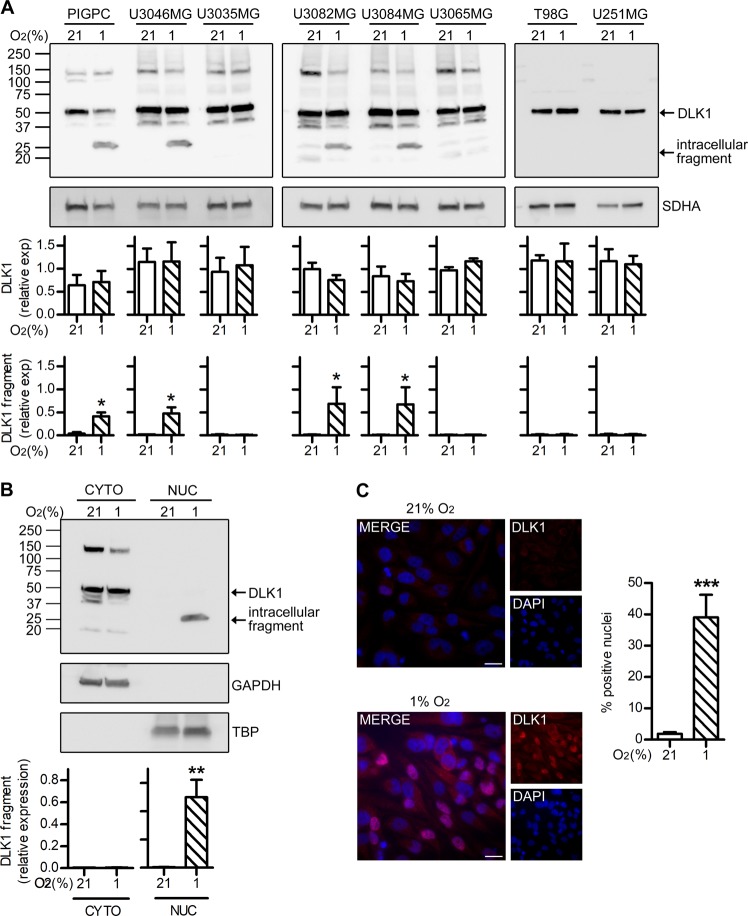
Hypoxia-induced cleavage and nuclear localization of DLK1 in GBM cells. **a** Representative images and densitometric analysis of western blots showing DLK1 expression and cleavage in different mouse (PIGPC) and human (U3046MG, U3035MG, U3082MG, U3084MG, U3065MG, T98G, and U251MG) GBM cell lines. Cells were grown at 21% or 1% O_2,_ as indicated, for 48 h. SDHA was used as loading control. **b** Representative images and densitometric analysis of western blots showing nuclear localization of a DLK1 intracellular fragment. Cellular fractionation experiments were performed in U3082MG grown at 21% or 1% O_2_ for 48 h. GAPDH and TBP were used as cytoplasmic and nuclear fractions controls, respectively. **c** Representative images and relative quantifications of immunofluorescent stainings showing DLK1 localization in U3082MG cells grown at 21% or 1% O_2_ for 48 h. Statistical analysis: independent experimental replicates are as follow, **a** for PIGPC, U3046MG, U3082MG, U3084MG, and U3065MG *n* = 4, for U035MG, U251MG, and T98G *n* = 3; in **b** *n* = 3 and in **c** *n* = 8. All data are expressed as mean ± SEM, statistical significance was determined by Mann–Whitney test applied to **a** and *t*-test with Welch’s correction applied to **b** and **c**. In the whole figure significance is represented as *p < 0.05, **p < 0.01, and ***p < 0.001 vs. respective 21% O_2_ controls.

We employed a panel of glioma cultures to characterize DLK1 under normoxic and hypoxic conditions. PDGFB-induced glioma primary cultures (PIGPC) and GBM cultures maintained under serum-free conditions (U3046MG, U3035MG, U3082MG, U3084MG, and U3065MG) [23] showed a band pattern similar to murine glioma (Fig. 2a), while serum-cultured cell lines T98G and U251MG showed a single DLK1 band (Fig. 2a). 4/6 glioma cultures showed lighter molecular weight band exclusive to hypoxic conditions (Fig. 2a). Since the antibody used detects a C-terminal DLK1 epitope, we hypothesized that hypoxia induced cleavage of an intracellular DLK1 fragment (Fig. 2a).

We evaluated the subcellular localization of DLK1 in U3082MG, U3084MG and PIGPC cells. Cellular fractioning experiments showed the presence of the intracellular fragment exclusively in the nuclear fraction (Fig. 2b, Supplementary Fig. 2A, B). These data were supported by immunofluorescent stainings, showing nuclear signal under hypoxia (Fig. 2c).

### DLK1 cleavage is dependent on HIFs and ADAM17

**Fig. 3:**
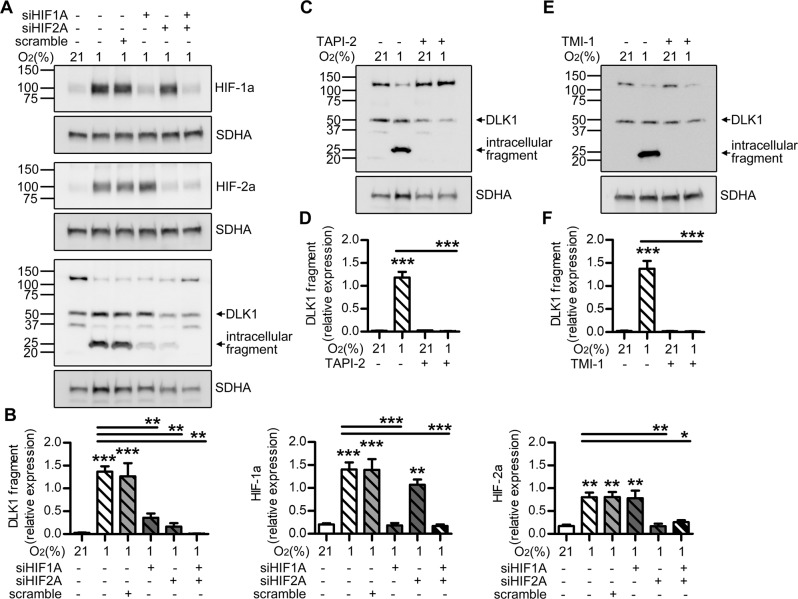
DLK1 cleavage is dependent on HIFs and ADAM 17 activity. **a**, **b** Representative images and densitometric analysis of western blots showing HIF-1α, HIF-2α, and DLK1 expression and cleavage in U3082MG cells after siRNA targeting of *HIF1A* and *HIF2A* in hypoxia. **c**, **d** Representative images and densitometric analysis of western blots showing the effects of ADAM inhibition by pre-treatment with 20 µM TAPI-2 on DLK1 cleavage in U3082MG cells grown at 21% or 1% O_2_ for 48 h. SDHA was used as loading control. **e**, **f** Representative images and densitometric analysis of western blots showing the effects of ADAM17 inhibition by pre-treatment with 0.5 μM TMI-1 on DLK1 cleavage in U3082MG cells grown at 21% or 1% O_2_ for 48 h. SDHA was used as loading control. Statistical analysis: **b** has 3 independent experiments while **d** and **f** have four independent experiments, all data are expressed as mean ± SEM. Statistical significance was determined by one-way ANOVA, followed by Bonferroni post hoc test. In the whole figure significance is represented as *p < 0.05, **p < 0.01, and ***p < 0.001 vs. respective 21% O_2_ controls or as indicated by straight lines.

We evaluated the role of HIF-induction on DLK1 cleavage using siRNA directed against *HIF1A*, *HIF2A*, or both. Knockdown of HIFs reduced DLK1 cleavage, with greater reduction by silencing *HIF2A*, and an almost complete abolition of cleavage by the combination of the two (Fig. 3a, b, Supplementary Fig. 3A, B).

Like canonical Notch ligands, DLK1 is a substrate of ADAM17 [12], an enzyme known to be hypoxia-induced in GBM [20]. Use of two independent ADAM17 inhibitors, TAPI-2 and TMI-1, blocked DLK1 cleavage (Fig. 3c–f, Supplementary Fig. 3C, D), suggesting that ADAM17-mediated cleavage of DLK1 precedes release of the intracellular fragment.

### Cleavable and uncleavable DLK1 forms differentially affect intracellular signaling pathways

**Fig. 4:**
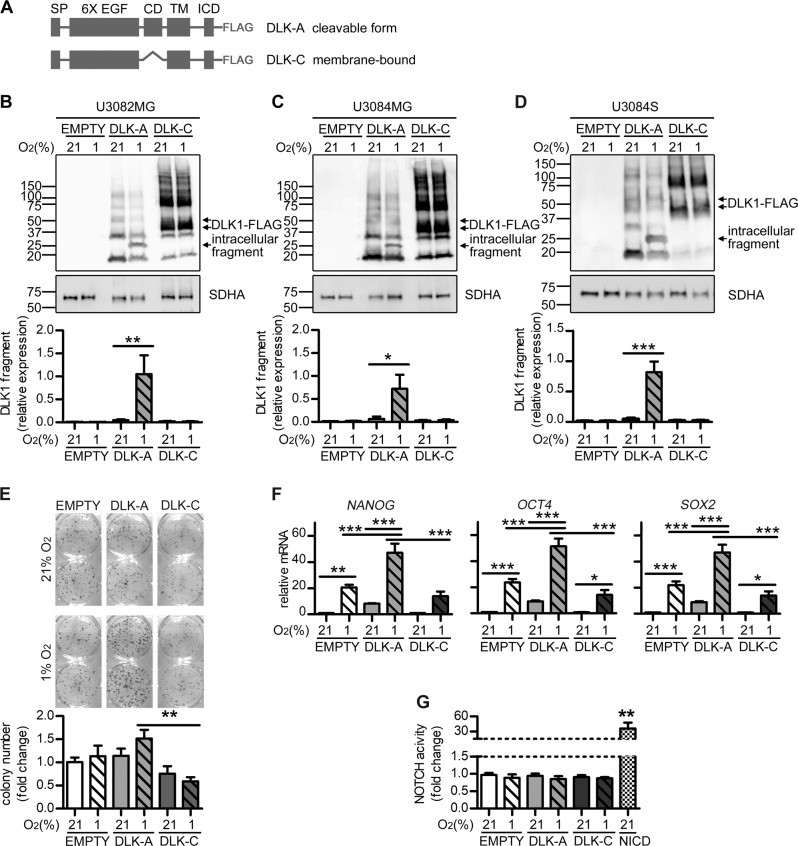
DLK1 cleavage influences stemness and clonal survival in stable cell lines. **a** Schematic representation of DLK1 cleavable (DLK-A) and uncleavable, membrane-bound (DLK-C) forms that were transiently or stably expressed in glioma cells. SP signal peptide, 6XEGF epidermal growth factor (EGF)-like repeats, CD extracellular cleavage domain, TM transmembrane domain, ICD intracellular domain, FLAG C-terminal tag. **b**, **c** Representative images and densitometric analysis of western blots showing FLAG-tagged DLK-A and DLK-C transient transfection experiments in U3082MG and U3084MG showing the lack of DLK-C cleavage in cells grown at 21% or 1% O_2_ for 48 h. SDHA was used as loading control. **d** Representative images and densitometric analysis of western blots showing U3084MG stable cell lines (U3084S) confirming FLAG-tagged DLKs stable expression and a similar behavior to the one obtained with transient transfection in U3084MG cells. SDHA was used as loading control. **e** Representative images and quantification of colony forming ability of U3084S stable lines grown in normoxic or hypoxic conditions. Data are expressed as fold change of normoxic EMPTY control. **f** qPCR data for relative mRNA expression of *NANOG*, *OCT4* and *SOX2* in U3084S cells grown at 21% or 1% oxygen for 48 h. Data are expressed as fold change of normoxic EMPTY control. **g** Dual luciferase assay data for NOTCH transcriptional activity in U3084S cells grown at 21% or 1% oxygen for 48 h. U3084MG cells transiently transfected with Notch ICD (NICD) were used as positive control. Data are expressed as fold change of normoxic EMPTY control. Statistical analysis: independent experimental replicates are as follow, **b** *n* = 4, **c** *n* = 3, **d** *n* = 6, **e** *n* = 4, **f** *n* = 5, and **g** *n* = 3. Statistical significance was determined by one-way ANOVA, followed by Bonferroni post hoc test. In the whole figure significance is represented as *p < 0.05, **p < 0.01, and ***p < 0.001 vs. respective 21% O_2_ controls or as indicated by straight lines.

We obtained two DLK1 constructs with a C-terminal FLAG-tag [11], DLK-A (full-length), and DLK-C (lacking the ADAM17 cleavage site; membrane bound) (Fig. 4a). We tested the behavior of the DLKs by transfection in U3082MG and U3084MG cells. In both lines DLK1 expressed with a pattern similar to the one we previously saw, with DLK-C exhibiting a stronger signal because of reduced degradation, as also demonstrated by the different intensity of a previously identified 12 kDa degradation product [24]. When cultured in hypoxia, DLK-A but not DLK-C presented cleavage in both lines (Fig. 4b, c). Detection of the fragment when blotting for the C-terminal FLAG-tag confirmed that the cleavage product represents the C-terminal end of DLK1.

We generated U3084MG cell lines stably expressing the two version of DLK1 (U3084S-DLK-A and U3084S-DLK-C). Cells maintained the same behavior observed in transient transfections, with DLK1 cleavage induced by hypoxia only in U3084S-DLK-A cells (Fig. 4d). U3084S-DLK-A cells displayed higher colony formation potential than U3084S-DLK-C or empty control cells at hypoxia (Fig. 4e). Moreover, U3084S-DLK-A cells presented higher levels of *NANOG*, *OCT4*, and *SOX2*, and the difference in expression levels was enhanced by hypoxia (Fig. 4f). The Notch pathway was unaffected by all DLK1 constructs at normoxia and hypoxia (Fig. 4g), as tested in a Notch luciferase reporter assay. These data were in agreement with a lack of correlation between levels of *DLK1* and Notch downstream effectors in human GBM, as analyzed in TCGA dataset (Supplementary Fig. 4). In addition, the DLK1 constructs did not affect hypoxia-induced cell death, as detected by propidium Iodide (PI) staining, and only moderately modulated caspase-3 activity (Supplementary Fig. 5A–C).

**Fig. 5:**
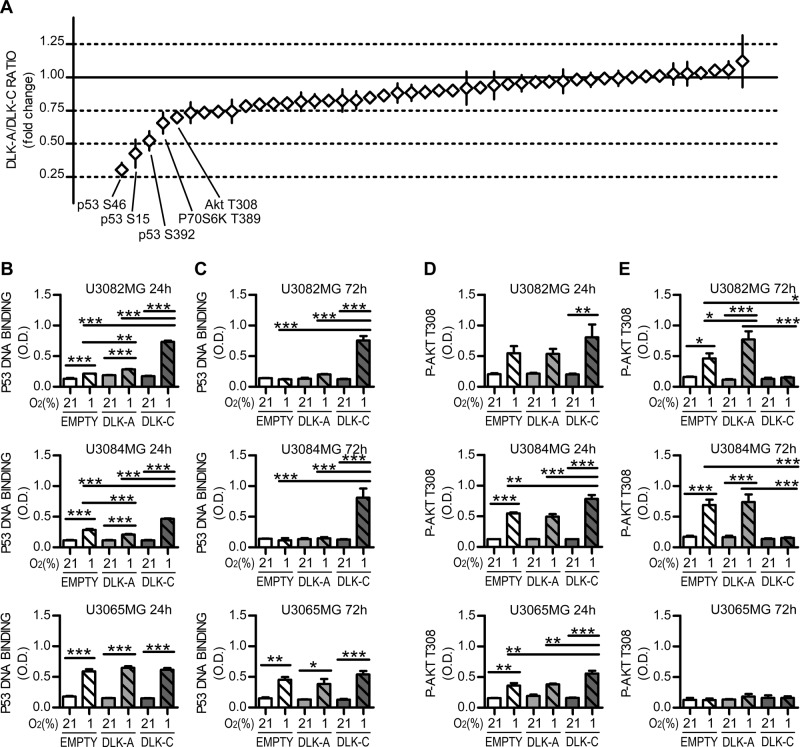
DLK1 cleavage influences p53 and Akt at hypoxia. **a** Graphical representation of Proteome profiler array results, showing variations of DLK-A vs. DLK-C kinase phosphorylation level ratio in relation to internal reference control (continuous line), in cells exposed to hypoxia for 24 h. Kinases with the most significant variations are highlighted in the figure. ELISA assay data showing p53 DNA binding activity in 3 different cell lines transiently transfected with DLKs, grown at 21% or 1% O_2_ for 24 (left, **b**) and 72 (right, **c**) h. **d**, **e** ELISA assay data showing Akt T308 phosphorylation in 3 different cell lines transiently transfected with DLKs, grown at 21% or 1% O_2_ for 24 (left, **b**) and 72 (right, **c**) h. Statistical analysis: all data are from three independent experiments and expressed as mean ± SEM, statistical significance was determined by one-way ANOVA, followed by Bonferroni post hoc test. In the whole figure significance is represented as *p < 0.05, **p < 0.01, and ***p < 0.001 as indicated by straight lines.

We measured variations in 45 phospho-kinases and 84 cancer-related proteins by proteome profiler arrays, comparing their levels in DLK-A versus DLK-C cells cultured in hypoxia. The results showed significant variations in levels of 43 proteins that are related to PI3K/Akt/mTOR pathway, p53-related stress and apoptosis, extracellular matrix degradation, and interleukin signaling (Supplementary Tables 1 and 2). We selected those that had a DLK-A/DLK-C expression ratio more significantly different than reference control for validation and further studies: p53 and Akt (Fig. 5a).

### DLK1 cleavage modulates cell metabolism under hypoxic conditions through the p53 and Akt pathways

Since the balance between Akt and p53 signaling is known to determine cell fate toward apoptosis or survival in hypoxia [25–27], we validated our results by p53 activity and Akt phosphorylation evaluation with ELISA assays, on three cell lines with different DLK1 cleavage abilities transfected with the various DLK1 constructs. All cell lines showed increased p53 DNA binding after 24 h of hypoxia, but at a later time point U3082MG and U3084MG cells had a persistent increase in DNA-binding ability only in cells transfected with DLK-C form, with levels back to basal in cells transfected with DLK-A or empty vector. By contrast, U3065MG, one of the cell lines that never showed DLK1 cleavage, showed a similar and persistent increase in DNA binding ability in all conditions and at a later time point (Fig. 5b, c). All cells showed increased Akt phosphorylation after 24 h of hypoxia exposure, with greater effects in DLK-C-transfected cells (Fig. 5d). At 72 h, however, U3084MG and U3082MG cells displayed greater phosphorylation levels detected in DLK-A-transfected cells and a return to basal levels in DLK-C-transfected cells (Fig. 5e). U3065MG showed increased Akt phosphorylation for all conditions at 24 h, but returned to basal level at 72 h (Fig. 5d, e). Taken together, these data indicate that DLK1 cleavage may influence the timing of p53 and Akt pathway activation after exposure to hypoxic conditions.

**Fig. 6:**
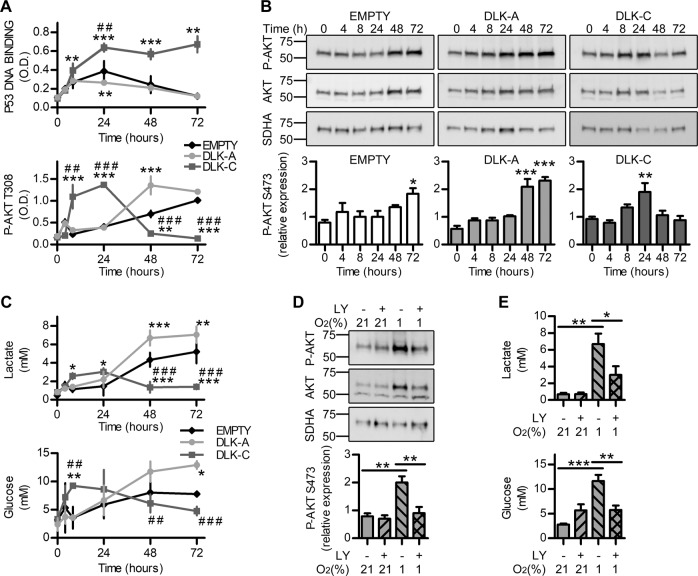
Temporal modulation of Akt and p53 balance by DLK1 cleavage regulates cell metabolism at hypoxia. **a** ELISA time course experiments showing p53 DNA-binding activity and Akt T308 phosphorylation variations in U3084S stable cell lines grown in 1% O_2_ for up to 72 h. **b** Western blot time course experiments showing variations in Akt S473 phosphorylation and total Akt levels in 3084 stable cell lines grown in 1% O_2_ for up to 72 h. SDHA was used as loading control. **c** Colorimetric assay time course experiment showing different modulation of glucose consumption and lactate production in 3084 S stable cell lines grown in 1% O_2_ for up to 72 h. **d** Representative images and densitometric analysis of western blots showing phosphorylated and total AKT levels in U3084S stable cell lines pre-treated for 24 h with 10 µM PI3K inhibitor LY294002 and then grown in 21% or 1% O_2_ for 48 h. SDHA was used as loading control. **e** Colorimetric assay experiment showing glucose consumption and lactate production variations in U3084S stable cell lines pre-treated for 24 h with 10 µM PI3K inhibitor LY294002 and then grown in 21% or 1% O_2_ for 48 h. Statistical analysis: all data are from four independent experiments, with the exception of point **c** with *n* = 3, and expressed as mean ± SEM. Statistical significance was determined by two-way ANOVA (**a**, **c**) and one-way ANOVA (**b**, **d**, **e**), followed by Bonferroni post hoc test. In the whole figure significance is represented as *p < 0.05, **p < 0.01, and ***p < 0.001 vs. EMPTY control or as indicated by straight lines, ^##^*p* < 0.01 and ^###^*p* < 0.001 of DLK-A vs. DLK-C.

We next evaluated p53 DNA binding ability and Akt phosphorylation at 0–72 h of hypoxia in U3084S lines. Results showed that U3084S-DLK-A and U3084S-DLK-C cells have different pathway activation kinetics at hypoxia. DLK-A overexpressing cells showed faster decline in p53 activation and stronger Akt T308 phosphorylation, while DLK-C overexpressing cells showed persistent p53 activation and only brief Akt phosphorylation (Fig. 6a). For further validation, we measured Akt S473 phosphorylation, which is usually required for full Akt activation [28]. Western blots showed stronger phosphorylation in U3084S-DLK-A cells at later time points and only a transient increase in U3084S-DLK-C cells (Fig. 6b). These results indicate that DLK1 cleavage may play a role in promoting cell survival in hypoxia, by induction of the Akt pathway and subsequent suppression of p53 activity. Since PI3K/Akt/mTOR pathways also play a role in metabolic reprogramming in GBM cells [29–31], we investigated whether that was the case in our setting. Indeed, U3084S cell lines showed significant differences in glucose consumption and lactate production in hypoxia. U3084S-DLK-A had significantly higher glucose consumption and lactate production (Fig. 6c), and inhibition of Akt phosphorylation by LY294002 (Fig. 6d) reverted the changes in glucose consumption and lactate production (Fig. 6e).

### DLK1 cleavage enhances GBM invasiveness in vitro and in vivo

**Fig. 7:**
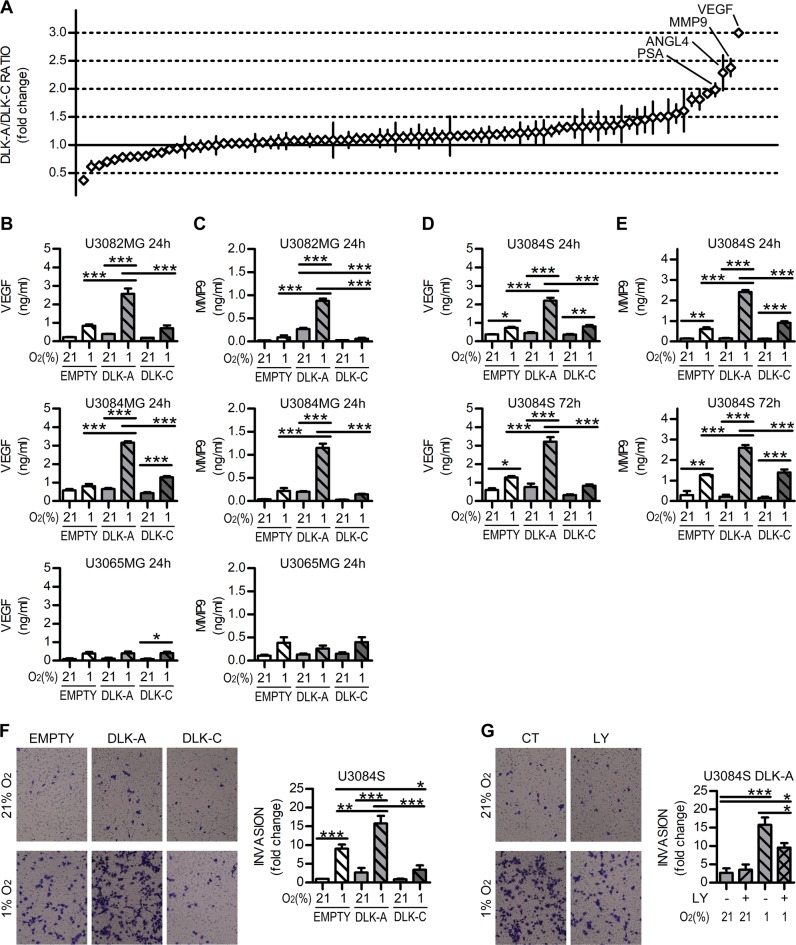
DLK1 cleavage enhances VEGF and protease secretion at hypoxia. **a** Graphical representation of Proteome profiler array results, showing variations of DLK-A vs. DLK-C cancer-related proteins levels ratio in respect to internal reference control (continuous line), in cells exposed to hypoxia for 24 h. Proteins with the most significant variations are highlighted in the figure. **b** ELISA assay data showing VEGF secretion in culture media in 3 different cell lines transiently transfected with DLKs, grown in 21% or 1% O_2_ for 24 h. **c** ELISA assay data showing MMP9 secretion in culture media in 3 different cell lines transiently transfected with DLKs, grown in 21% or 1% O_2_ for 24 h. **d** ELISA assay data showing VEGF secretion in culture media in U3084S stable cell lines, grown in 21% or 1% O_2_ for 24 and 72 h. **e** ELISA assay data showing MMP9 secretion in culture media in U3084S stable cell lines, grown in 21% or 1% O_2_ for 24 and 72 h. **f** Representative images and quantification of transwell matrix invasion assay showing invasion ability of U3084S stable cell lines, grown in 21% or 1% O_2_ for 24 h. Data are expressed as fold change of normoxic EMPTY control. **g** Representative images and quantification of transwell matrix invasion assay showing invasion ability of U3084S-DLK-A cells pre-treated with 10 µM PI3K inhibitor LY294002 for 24 h and then grown in 21% or 1% O_2_ for 24 h. Data are expressed as fold change of normoxic EMPTY control. Statistical analysis: all data are from three independent experiments, with the exception of point **f** with *n* = 4, and expressed as mean ± SEM. Statistical significance was determined by one-way ANOVA followed by Bonferroni post hoc test. In the whole figure significance is represented as *p < 0.05, **p < 0.01, and ***p < 0.001 as indicated by straight lines.

We then validated proteome array data on VEGF and MMP9, the two proteins with the greatest difference between DLK-A and DKL-C (Fig. 7a). We used ELISA assays to measure the amount of MMP9 and VEGF in cell culture supernatant in normoxia and after 24 h of hypoxia. We obtained significantly greater increase of both proteins in DLK-A transfected U3082MG and U3084MG cells, while no differences were detected in U3065 cells that do not show DLK1 cleavage (Fig. 7b, c). Similar results were obtained in U3084S lines, with significantly higher VEGF and MMP9 production in U3084S-DLK-A cells exposed to hypoxia. These differences were maintained also at later timepoint (Fig. 7d, e).

Since many of the proteins with significant variations detected in the proteome profiler array are involved in extracellular matrix remodeling and degradation, we investigated whether expression of the various DLK1 forms could influence the invasive behavior of glioma cells. Experiments on matrix-coated transwell chambers revealed that, after hypoxia exposure, U3084S-DLK-A cells had a significantly higher invasion potential as compared with controls, while U3084S-DLK-C ones showed a significant reduction in invasion (Fig. 7f). As PI3K/Akt pathway has been reported to be involved in cancer cell invasiveness and MMP9 and VEGF regulation [32, 33], we tested the involvement of this pathway in our system. Indeed, the use of the LY294002 significantly reduced transwell invasion in U3084S-DLK-A cells (Fig. 7g).

To test whether the effects of DLK-A overexpression described here were induced by release of the intracellular fragment rather than the extracellular domain, we overexpressed a DLK1 construct constituted only by the cleaved extracellular domain (DLK-S) (Supplementary Fig. 6A). DLK-S did not induce any significant change in VEGF or MMP9 levels, and the invasion potential of U3084MG cells was unaffected by DLK-S expression (Supplementary Fig. 6B–D).

**Fig. 8:**
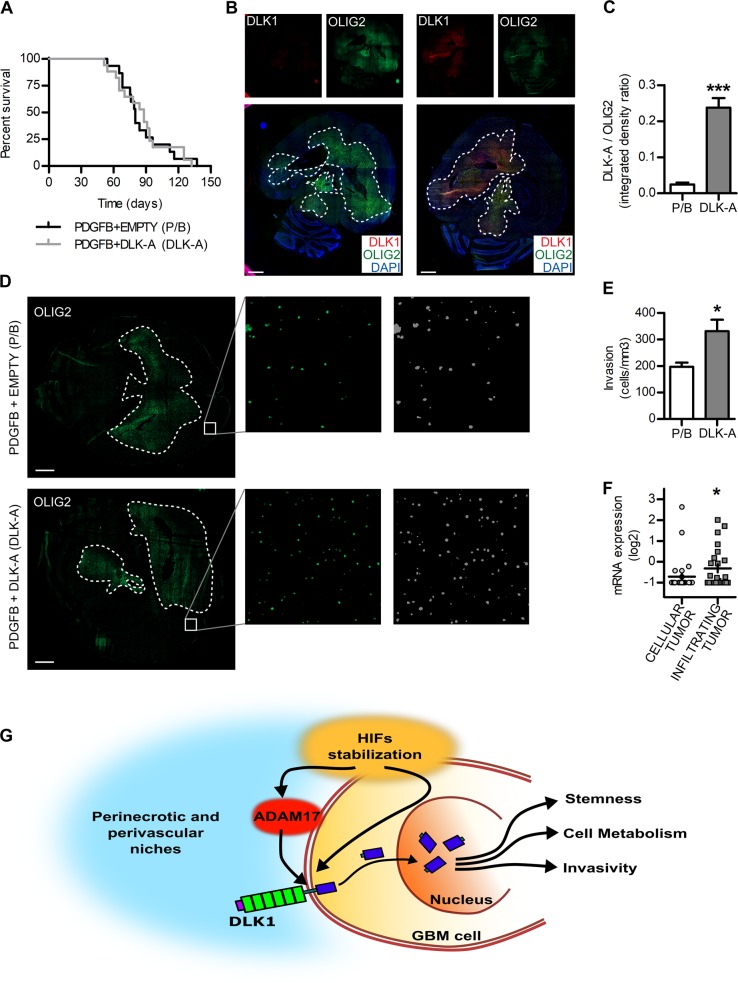
DLK1-expressing gliomas are more invasive in vivo. **a** Kaplan–Meier survival plot of PDGFB-induced tumors with (DLK-A, gray line) or without (P/B, black line) DLK1 overexpression. **b**, **c** Representative images and signal intensity quantification of immunofluorescent stainings showing DLK1 expression levels in brains with PDGFB and DLK-A gliomas. OLIG2 was used as a marker of tumor area and as signal intensity normalizer. Dashed line encircles tumor area, as defined by OLIG2 staining. **d**, **e** Representative images and signal intensity quantification of immunofluorescent stainings showing presence of OLIG2 positive cells outside the bulk tumor in P/B and DLK-A gliomas. Inserts show representative magnified images of the random fields and corresponding thresholded overlay used for cell count. Dashed line encircles tumor area, as defined by OLIG2 staining. **f** Dotplot showing relative mRNA expression levels of *DLK1* in microdissected cellular tumor versus infiltrating tumor cells from the Ivy GAP RNA-sequencing dataset. Scale bar represent 1 mm. **g** Graphical representation of DLK1 processing and its effects in perinecrotic and perivascular niches. Statistical analysis: Kaplan–Meier survival plot shows a total of 32 experimental mice of which 15 in P/B group and 17 in DLK-A group. **b**–**e** These were obtained from analysis of seven brains for each group, and statistical significance determined by *t*-test. For **f**, 54 tumor samples (cellular = 30, infiltrating = 24) were analyzed with GlioVis tool and statistical significance determined by *t*-test. In all the figure significance is represented as *p < 0.05 and ***p < 0.001.

To test the effects of DLK1 expression in vivo, we used the RCAS/tv-a system to overexpress *PDGFB* in combination with DLK-A in Ntv-a mice. There was no difference in survival between PDGFB controls and PDGFB + DLK-A mice (Fig. 8a). Enhanced DLK1 levels in PDGFB + DLK-A tumors were confirmed by co-staining with the tumor marker Olig2 (Fig. 8b, c). We examined the presence of tumor cells in randomly selected areas outside of the tumor bulk, to investigate whether the enhanced invasive behavior of DLK-A-expressing cells in vitro was reflected in invasive behavior in vivo. Indeed, we found significantly higher numbers of Olig2+ cells outside the tumor bulk in PDGFB + DLK-A brains compared with controls (Fig. 8d, e), indicating that DLK-A may drive invasive tumor growth. Analysis of DLK1 expression in the Allen Institute for Brain Science Ivy Glioblastoma Atlas Project (Ivy GAP) database [34] revealed increased expression of DLK1 in microdissected areas of infiltrating tumor as compared with in the cellular tumor bulk (Fig. 8f), suggestive of a significant role for DLK1 in the invasive behavior of GBM cells.

## Discussion

Release of the extracellular domain of DLK1 mediated by ADAM17 is central to paracrine signaling by DLK1 [12]. Many substrates of ADAM17 undergo further intracellular processing following ADAM17-mediated cleavage to release intracellular domains with nuclear signaling roles. This is true for Notch receptors and canonical Notch ligands such as Dll1 and Jag1 [35–37]—some of the closest relatives to DLK1—and for proteins involved in brain tumor and neural stem cell maintenance like CD44 [20, 38, 39] and p75NTR [40, 41]. While DLK1 is equipped with an intracellular domain, little data have supported an intracellular role for DLK1, and DLK1 signaling has largely been assumed to be mediated by the extracellular domain. Our data show that ADAM17-mediated DLK1 cleavage is followed by the release and nuclear translocation of a C-terminal DLK1 fragment. The identity of the fragment remains to be determined: the predicted molecular weight of the DLK1 intracellular domain is half of the fragment we identify. The facts that the fragment is recognized only by antibodies targeted to C-terminal DLK1, and includes the C-terminal tag in our constructs, indicate that the intracellular domain is part of the nuclear fragment, suggesting that the higher-than-anticipated molecular weight might be due post-translational modifications. The release of this intracellular fragment appears necessary for many of the biological consequences associated with DLK1 expression, as the non-cleavable DLK-C construct and the ECD-only DLK-S construct were unable to mediate the same metabolic and migratory properties in our models.

The best-described mechanisms of action for DLK1 have been in regulation of Notch signaling, where contradictory findings have indicated that DLK1 can act to inhibit [42] or promote [43] Notch signaling. Our findings join a third category of reports suggesting that DLK1 effects are independent on Notch: DLK1-overexpressing cells were no different in their activation of Notch signaling compared with controls in a reporter assay, and DLK1 expression in no way correlated with expression of classical Notch downstream target genes in human GBM, as analyzed in TCGA data. In spite of the close structural relation between DLK1 and canonical Notch ligands, mechanisms of DLK1 signaling in glioma appear to be more complex than inhibition of Notch receptor activation.

Our findings add to a growing literature describing hypoxia-induced activation of ADAM17-dependent proteolytic cleavage of proteins promoting aggressive tumor cell properties like stemness and invasive growth in brain tumors and other cancer forms [20, 41, 44]. In culture, the nuclear localization of DLK1 was exclusive to cells cultured in hypoxia. In vivo, however, we detected DLK1 in the nucleus not only in hypoxic tumor areas, but also in the perivascular niche. These data are in agreement with our previous observations that tumor cells of the perivascular niche display a pseudo-hypoxic phenotype driven by high expression levels of HIF-2alpha in these presumably well-oxygenated tumor areas [20, 45]. It is likely that ADAM17 can become activated under other HIF-independent circumstances, suggesting that DLK1 cleavage and nuclear translocation may occur in other contexts as well. DLK1 has previously been associated with hypoxia in other tumor types [6], suggesting that DLK1 expression and activity may both be driven by hypoxia-dependent mechanisms. The mechanisms involved in regulating DLK1 cleavage by ADAM17 appear to be complex, as we did not detect the intracellular fragment in four tested glioma cell lines in hypoxia. ADAM17 activity itself could be differentially regulated in these cells, and it is possible that the availability and abundance of other ADAM17 substrates could affect the likelihood of DLK1 cleavage.

In the model systems investigated here, DLK1 overexpression was not sufficient to drive a lower survival rate of glioma-bearing mice, but tumors generated to express DLK1 did show a more invasive growth pattern. Other hypoxia-activated ADAM17 substrates have previously been shown to accelerate tumor progression in mouse models of glioma. Whether or not these proteolytic events can be therapeutically targeted remains to be investigated.

In conclusion, we found an unexpected nuclear localization of DLK1 in hypoxic glioma cells, and demonstrated that a C-terminal fragment of DLK1 has signaling activity resulting in altered p53 and PI3K pathway activation (Fig. 8g). Further work is required to characterize the identity and function of the intracellular fragment in greater detail.

## Materials and methods

### Generation of murine gliomas

Gliomas were induced in neonatal Nestin-tv-a (Ntv-a) or Ntv-a Ink4a/Arf^−/−^ mice by intracranial injection of RCAS-PDGFB, RCAS-shp53, or RCAS-cre transfected DF1 cells [19, 46].

DLK-A was cloned in the RCAS vector and all pups of each litter were injected with a 1:1 mix of DF1 cells expressing PDGFB and DLK-A or empty vector. Each litter was allocated to one experimental group. Mice were monitored daily and euthanized at the onset of glioma symptoms. All procedures were approved by Malmö-Lund Ethical Committee (M186–14).

Minimal sample number was determined based on law of diminishing returns with the resource equation method (total number of animals - total number of groups >10). A total of 8 pups were excluded due to non-tumor symptoms during week 0–3, final numbers: *n* = 15 PDGFB and *n* = 17 DLKA.

### Western blot

Cells were lysed in RIPA buffer with Complete phosphatase and Protease inhibitor cocktails (Roche, Basel, Switzerland). Cellular fractionation was performed with NE-PER Nuclear and Cytoplasmic Extraction Kit (ThermoFisher, Waltham, MA). Tissue lysates were prepared as previously reported [41]. Equal amounts of samples were diluted in DTT Laemmly buffer, boiled 5 min and loaded on 4–20% Mini-PROTEAN^®^ TGX™ Precast Protein Gels (Biorad, Hercules, CA), transferred on PVDF membranes with Transblot Turbo System (Biorad), blocked in 5% milk/TBS-T and incubated overnight at 4 °C with primary antibodies. Membranes were washed and incubated for 1 h with the secondary antibodies (Abcam, Cambridge, UK), developed with Luminata Forte western HRP Substrate (Millipore, Burlington, MA) and images acquired with a Fujifilm LAS 3000 Imager (Fujifilm, Tokio, Japan).

Primary antibodies: DLK1 ab21682 (Abcam), SDHA ab14715 (Abcam), GAPDH 3683 (Cell Signaling), TATA binding protein TBP ab51841 (Abcam), HIF-1a NB100–479 (Novus Biologicals), HIF-2a ab199 (Abcam), FLAG F1804 (Sigma), Akt Ser473 OMA1–03061 (ThermoFisher), pan-AKT ab38449 (Abcam).

Band intensity was quantified using ImageJ and three different exposures/membranes were averaged for each independent experiment.

For tissue samples, *n* = 3. Hypoxia experiments *n* = 4 except in U3035MG, T98G, and U251MG *n* = 3. Cellular fractionation and HIF silencing experiments *n* = 3. ADAM17 inhibition *n* = 4. DLK1 overexpression, U3082MG *n* = 4, in U3084MG *n* = 3. U3084S stable lines *n* = 6 for DLK1, *n* = 4 for Akt.

### Immunofluorescence

Brains were collected, embedded in OCT (ThermoFisher), and frozen in pre-cooled. Cryosections (5 µm) were air-dried for 30 min, fixed in ice-cold acetone and permeabilized in 0.3% Triton X-100/PBS (Sigma). Sections were blocked in serum-free protein block (DAKO) and incubated overnight at 4 °C with primary antibodies diluted in antibody diluent with Background Reducing Components (DAKO). AlexaFluor secondary antibodies (Abcam) were used and slides mounted in Vectashield Mounting medium with DAPI (Vector Laboratories).

Cells were grown on polyornithyne/laminin coated glass slides, fixed 10 min in 4% PFA and permeabilized with 0.2% Saponin/PBS (Sigma). Slides were blocked in 5% BSA/PBS for 1 h and incubated overnight at 4 °C with primary antibodies diluted in 5% BSA/PBS. Slides were incubated with AlexaFluor secondary antibodies diluted in 5% BSA/PBS, and mounted in Vectashield Mounting medium with DAPI.

Primary antibodies: DLK1 NBP2–33697 (Novus Biologicals), DLK1 PA5–72199 (ThermoFisher), DLK1 LS‑C179442 (LSBio), HIF-1a NB100–479 (Novus Biologicals), CD44 550538 (BD Biosciences), OLIG2 AF2418 (R&D Systems). Images were acquired using an Olympus BX63 microscope and DP80 camera and cellSens Dimension v 1.12 software (Olympus Corporation, Tokio, Japan).

Staining was quantified using ImageJ. For tissue slides, DLK1 quantification was performed on whole slide of three independent brains. Nuclear intensity and nuclear percentage of DLK1 positive cells were quantified by analyzing three regions per tumor, total nuclei analyzed: perinecrotic *n* = 872, bulk *n* = 785, perivascular *n* = 743, bulk *n* = 847. DLK1 expression in different grades: four brains/group were analyzed, with quantification of four fields each. DLK-A overexpression: quantification was performed on whole slide acquisition of seven brains per group. Cell experiments: four independent experiments were performed in duplicate, total number of nuclei 1177 (control), 1106 (hypoxia).

### Cell culture and cell treatments

PIGPCs were isolated as previously described [39] and grown in DMEM (Life Technologies) with 10% fetal bovine serum (FBS) and 1% PenStrep solution (Corning). U3046MG, U3035MG, U3082MG, U3084MG, and U3065MG cells were obtained from the Human Glioblastoma Cell Culture Resource (HGCC) and cultured as described previously [23] in Neurobasal (GIBCO) and DMEM/F12 with Glutamax media (Life Technologies), 1:1 mix, with 1% PenStrep, N2 and B27 (Life Technologies), 10 ng/mL epidermal growth factor (EGF), and 10 ng/mL fibroblast growth factor (FGF) (Peprotech). Cells were dissociated by Accutase (ThermoFisher) treatment, and grown as a monolayer on plastic dishes coated with polyornithine (Sigma) and laminin (Biolamina). U251MG and T98G cells were obtained from ATCC and grown in DMEM with 10% FBS and 1% PenStrep solution. Cells were used within ten passages. Mycoplasma contamination was tested every 3 months.

Hypoxia (1%O_2_) was generated in a Whitley H35 Hypoxystation (Don Whitley Scientific, Bingley, UK).

Reagents: TAPI-2 (Sigma), MI1 (Tocris Bioscience), LY294002 (Sigma), added 24 h pre-hypoxia exposure.

### Transient transfection, luciferase assay, and stable line selection

DLK plasmids were a kind gift from Ferguson et al. [11]. Plasmids were transfected using Xtreme gene 9 (Roche) and siRNAs using HiPerFect (QIAGEN). Non-targeting (D-001810–01–20) HIF1A (LQ-004018–00–0002) and HIF2A (LQ-004814–00–0002) siRNAs were obtained from GE Dharmacon. For the generation of U3084S stably expressing DLKs, U3084MG cells were transfected, selected using 750 µg/ml G418 and expanded. Stable pools were used without cloning.

For luciferase reporter assays, cells were co-transfected with 8xCSL-luc (gift from Håkan Axelson) and pCMV-renilla (Promega), together with indicated constructs, then analyzed using the Dual-Luciferase Reporter Assay System (Promega) on a Synergy 2 platereader (BioTek, Winooski, USA). The human Notch1-ICD plasmid was a gift from Håkan Axelson.

For Notch luciferase assay, three independent experiments were performed, read in duplicate.

### Colony formation assay

Single cell suspension was prepared by mechanical dissociation after Accutase (ThermoFisher) treatment and cells were seeded at 150 cells/well, in six-well plates. Cells were grown for 2 weeks, washed with PBS, fixed with 4% PFA, and stained in crystal violet. Images were acquired with Fujifilm LAS 3000 Imager. Four independent experiments were performed in duplicate.

### Real-time qPCR

RNA was isolated using the RNeasy Mini Kit and the Qiashredder Kit (QIAGEN) according to manufacturer instructions, and cDNA synthesized using random primers and Multi-Scribe reverse transcriptase enzyme (Applied Biosystems). The amplifications were run using a QuantStudio 7 real-time PCR system (Applied Biosystems, Foster City, USA) with SYBR Green Master Mix (Applied Biosystems). Relative gene expression was normalized to the expression of three housekeeping genes (UBC, SDHA, and YWHAZ) using the comparative ΔΔCT method. Five independent experiments were performed, read in duplicate.

Primers:

**Table:** 

	FORWARD	REVERSE
***NANOG***	GCTGGTTGCCTCATGTTATTATGC	CCATGGAGGAAGGAAGAGGAGAGA
***SOX2***	GCCTGGGCGCCGAGTGGA	GGGCGAGCCGTTCATGTAGGTCTG
***OCT4***	AGCAAAACCCGGAGGAGT	CCACATCGGCCTGTGTATATC
***UBC***	ATTTGGGTCGCGGTTCTT	TGCCTTGACATTCTCGATGGT
***SDHA***	TGGGAACAAGAGGGCATCTG	CCACCACTGCATCAAATTCATG
***YWHAZ***	ACTTTTGGTACATTGTGGCTTCAA	CCGCCAGGACAAACCAGTAT

### Cell death evaluation

Cell death was determined by PI staining. Cells were exposed to normoxia or hypoxia for 4 or 72 h. After dissociation, cells were collected and centrifuged for 5 min at 300 × *g*. The pellet was washed once with PBS and resuspended in FACS buffer (PBS, 2% FBS, 1 mM EDTA, 0.1% sodium azide) containing 0.05 μg/ml PI. Cells were analyzed using FACSVerse instrument (BD, Franklin Lakes, USA) and data analyzed with FlowJo software.

Caspase-3 activity was measured with Caspase-3 Assay Kit ab39401 (Abcam) following manufacturer’s instruction.

All experiments were performed in triplicate.

### Proteome profiler array and validation experiments

Eighty-four different cancer-related proteins and the 43 kinases phosphorylation were measured with Proteome Profiler Human XL Oncology Array and Proteome Profiler Human Phospho-Kinase Array Kit (R&D Systems). Membranes were developed with Luminata Forte western HRP Substrate (Millipore) and images acquired with a Fujifilm LAS 3000 Imager. Dot intensity was quantified with ImageJ. Values were normalized to internal reference control. A restricted number of candidates were selected by plotting the DLK-A/DLK-C ratio of variation vs. internal reference control.

p53 activity was measured on nuclear extracts with p53 Transcription Factor Assay Kit (Abcam ab207225) following manufacturer’s instruction; Akt phosphorylation levels were measured with AKT1 + AKT2 + AKT3(pT308) ELISA Kit (ab176636) on total cellular extractions; Glucose and Lactate levels, VEGF and MMP9 secretion were measured on supernatant media with Glucose Assay Kit ab102517 (Abcam), L-Lactate Assay Kit (Abcam ab65331), Human VEGF-A ELISA Kit RAB0507 (Sigma) and Human MMP9 ELISA Kit ab100610 (Abcam). Each sample was read in duplicate. For p53, P-Akt, VEGF, MMP9 *n* = 3 for U3082MG, U3084MG, U3065MG. U3084S, *n* = 4 (p53, P-Akt), *n* = 3 (VEGF, MMP9, lactate, glucose).

### Invasion quantification

Transwell migration assays were performed in Matrigel-coated Transwell, 8 µm pore size (Corning). 5 × 10^5^ cells/mL were resuspended in DMEM/F12 medium and 100 µL were transferred into the upper chamber. After 6 h, growth factors were added as chemoattractants in the lower chamber and cells placed at 21 or 1% O_2_. After 24 h, transwells were washed in PBS and fixed in 4% PFA. Non-migrating cells on the upper surface were removed, and remaining cells on the bottom were stained with crystal violet. Ten random fields per well were acquired using an Olympus BX63 microscope, DP80 camera, and cellSens Dimension 1.12 software (Olympus Corporation). Cells were counted with ImageJ. *N* = 4 (stable line comparison), *n* = 3 (Akt inhibition)

For in vivo invasion, seven brains per group were stained with OLIG2 to identify cancer cells. OLIG2+ cells were counted in five fields selected at least 500 µM from tumor bulk border.

### Statistical analyses

Data from The Cancer Genome Atlas (TCGA, https://portal.gdc.cancer.gov/), the Chinese Glioma Genome Atlas (CGGA, http://www.cgga.org.cn/) and Allen Institute for Brain Science Ivy Glioblastoma Atlas Project (IvyGap, http://glioblastoma.alleninstitute.org) were analyzed using GlioVis [47]. For DLK1 expression, data from 620 TCGA patients (grade II *n* = 226, grade III *n* = 244, grade IV *n* = 150), from 651 CGGA patients (grade II = 232, grade III = 194, grade IV = 225) and from 54 IvyGap tumor samples (cellular = 30, infiltrating = 24) were analyzed. Tukey’s HSD test was used to determine statistical significance. For *DLK1* correlation with *HEY1*, *HEY2* and *HES1* expression 160 TCGA samples were analyzed and significance determined with Pearson’s method.

Experiments and analyses were not performed in blind. Samples were not randomized to experimental groups. After normal distribution and variance similarity evaluation, two-sided unpaired *t*-test (eventual Welch’s correction for groups with different variances), Mann–Whitney for non-parametric data, one-way ANOVA with Bonferroni post-hoc test and two-way ANOVA (timelines only) tests were used to determine statistical significance, as indicated in respective figure legends. Data largely met the assumptions of the indicated statistical tests. For survival evaluation, the Kaplan–Meier method was used to investigate variables and overall survival correlation, while a log-rank test was employed to compare survival curves. In all figures data are shown as mean ± SEM, analyzed using GraphPad Prism 5 software and significance expressed as *p* values (*p < 0.05, **p < 0.01, ***p < 0.001).

## Supplementary information

Suppl. Figure and Table Legends

Suppl. Fig. 1

Suppl. Fig. 2

Suppl. Fig. 3

Suppl. Fig. 4

Suppl. Fig. 5

Suppl. Fig. 6

Suppl. Table 1

Suppl. Table 2
