# Supplementary material for: Hypoxia-induced release, nuclear translocation, and signaling activity of a DLK1 intracellular fragment in glioma
Source: Oncogene. 2020 Mar 24;39(20):4028–44. doi: 10.1038/s41388-020-1273-9 (PMC7220882; doi:10.1038/s41388-020-1273-9)
Supplement: Supplementary file 1 — Suppl. Figure and Table Legends [file 41388_2020_1273_MOESM1_ESM.docx]

**Supplemental Table 1**

Proteome Profiler Human Phospho-Kinase Array data expressed as Intensity normalized by internal reference control. Statistical significance was determined by t-test. * p<0.05, ** p<0.01, *** p<0.001.

**Supplemental Table 2**

Proteome Profiler Human XL Oncology Array data expressed as Intensity normalized by internal reference control. Statistical significance was determined by t-test . * p<0.05, ** p<0.01, *** p<0.001.

**Supplemental Figure 1**

A, B: Representative images and signal intensity quantification of immunofluorescent stainings showing DLK1 expression levels in brains with PDGFB-induced low grade and PTEN loss or shp53-induced high grade murine gliomas. Dashed line encircles tumor area. Scale bars represent 1 mm.

C: Boxplots showing DLK1 expression levels in 651 human gliomas from CGGA database, stratified by tumor grade.

Statistical analysis: four brains were analyzed for each glioma grade and for each tumor four areas of tumor and healthy brain were analyzed. Data are expressed as mean±SEM and statistical significance was determined by Mann-Whitney test. For CGGA dataset, statistical significance was determined by Tukey’s HSD using the GlioVis tool. In the whole figure significance is represented as * p<0.05, **p<0.01 and *** p<0.001.

**Supplemental Figure 2**

A, B: Representative images and densitometric analysis of western blots showing nuclear localization of a DLK1 intracellular fragment. Cellular fractionation experiments were performed in U3084MG (A) and PIGPC (B) cells grown at 21% or 1% O_2_ for 48 hours. GAPDH and TBP were used as cytoplasmic and nuclear fractions controls, respectively.

Statistical analysis: all experiments were performed in triplicate and all data are expressed as mean±SEM. Statistical significance was determined by Mann-Whitney test. In the whole figure significance is represented as * p<0.05 and *** p<0.001 vs. respective 21% O_2_ controls.

**Supplemental figure 3**

A,B: Representative images and densitometric analysis of western blots showing HIF-1a, HIF-2a and DLK1 expression and cleavage in U3084MG cells after siRNA targeting of *HIF1A* and *HIF2A* in hypoxia.

C,D: Representative images and densitometric analysis of western blots showing the effects of ADAM inhibition by pre-treatment with 20µM TAPI-2 on DLK1 cleavage in U3084MG cells grown at 21% or 1% O_2_ for 48 hours.

SDHA was used as loading control.

Statistical analysis: in image A data are from three independent experiments while in B data are from 3 independent experiments. In all figure data are expressed as mean±SEM and statistical significance was determined by one-way ANOVA, followed by Bonferroni post hoc test. Significance is represented as * p<0.05, ** p<0.01 and *** p<0.001 vs. respective 21% O_2_ controls or as indicated by straight lines.

**Supplemental Figure 4**

Scatterplot showing lack of correlation in the levels of DLK1 and Notch downstream effectors *HEY1*, *HEY2* and *HES1* mRNA levels in TCGA dataset.

Statistical analysis: 160 tumor samples were analyzed with GlioVis tool and significance assessed with Pearson’s correlation method.

**Supplemental Figure 5**

A: Representative images and relative quantifications of live cells as determined by Propidium Iodide staining of U3084S stable lines cells grown at 21% or 1% O_2_ for 4 hours.

B: Representative images and relative quantifications of live cells as determined by Propidium Iodide staining of U3084S stable lines cells grown at 21% or 1% O_2_ for 72 hours.

C: Caspase-3 activity in U3084S stable lines, U3082MG, U3084MG and U3065MG cells grown at 21% or 1% O_2_ for 72 hours.

Statistical analysis: all data are from three independent experiments, and expressed as mean±SEM. Statistical significance was determined by one-way ANOVA followed by Bonferroni post hoc test. In the whole figure significance is represented as * p<0.05, ** p<0.01 and *** p<0.001 as indicated by straight lines.

**Supplemental Figure 6**

A: Schematic representation of DLK1 cleavable (DLK-A) and extracellular domain secreted (DLK-S) forms that were stably expressed in glioma cells. SP, signal peptide; 6XEGF, epidermal growth factor (EGF)-like repeats; CD, extracellular cleavage domain; TM, transmembrane domain; ICD, intracellular domain; FLAG, C-terminal tag; ECD extracellular domain.

B: ELISA assay data showing VEGF secretion in culture media in U3084S stable cell lines, grown in 21% or 1% O_2_ for 24 and 72 hours.

C: ELISA assay data showing MMP9 secretion in culture media in U3084S stable cell lines, grown in 21% or 1% O_2_ for 24 and 72 hours.

D: Representative images and quantification of transwell matrix invasion assay showing invasion ability of U3084S stable cell lines, grown in 21% or 1% O_2_ for 24 hours. Data are expressed as fold change of normoxic EMPTY control.

Please not that throughout this figure, DLK-A and EMPTY data are the same as data presented in Fig. 7, presented here for comparison with DLK-S as they were all performed in the same experiments.

Statistical analysis: all data are from three independent experiments, with the exception of point D with n=4, and expressed as mean±SEM. Statistical significance was determined by one-way ANOVA followed by Bonferroni post hoc test. In the whole figure significance is represented as * p<0.05, ** p<0.01 and *** p<0.001 as indicated by straight lines.
