# Supplementary figures and images for: Hypoxia-induced release, nuclear translocation, and signaling activity of a DLK1 intracellular fragment in glioma
Source: Oncogene. 2020 Mar 24;39(20):4028–44. doi: 10.1038/s41388-020-1273-9 (PMC7220882; doi:10.1038/s41388-020-1273-9)

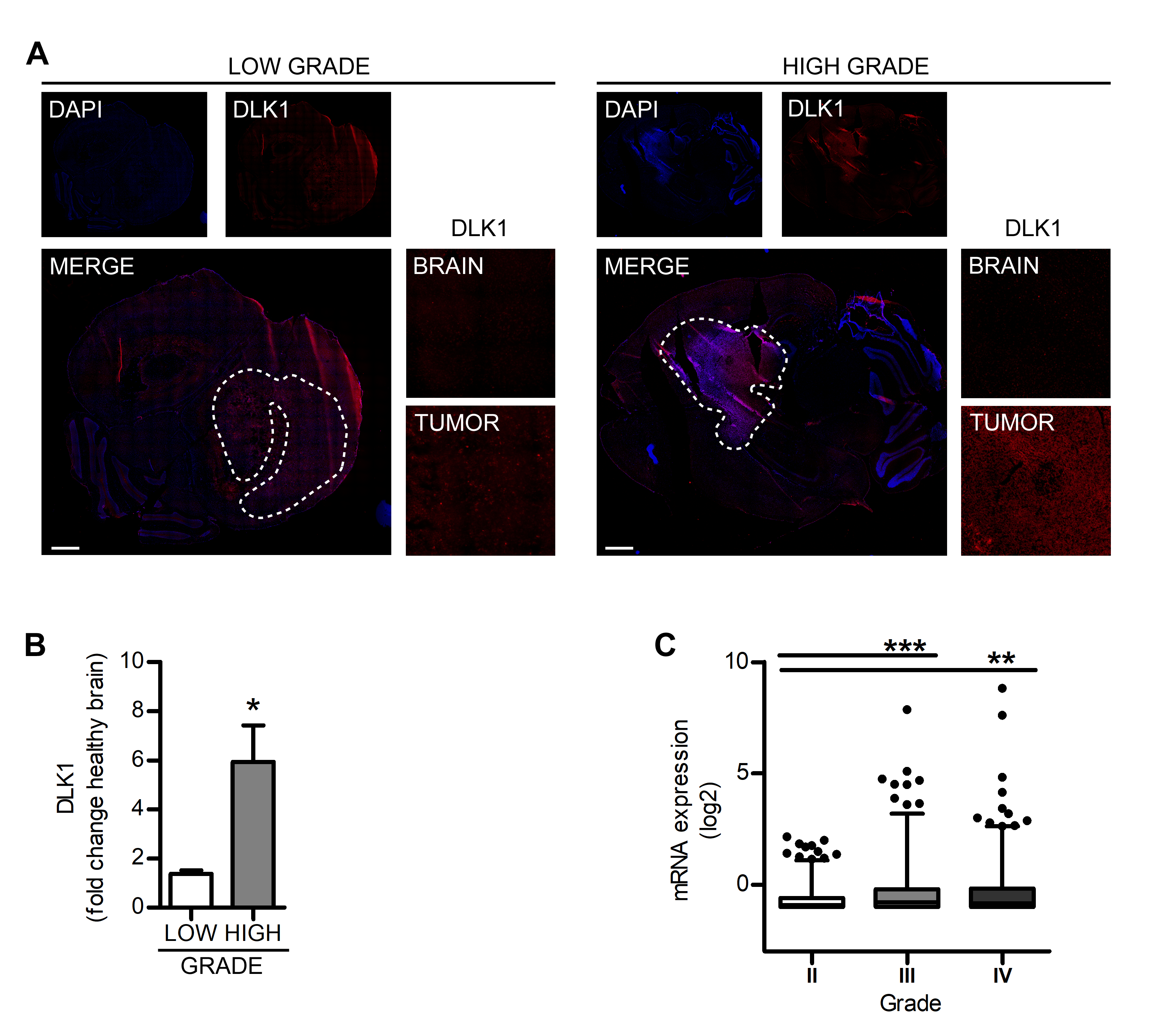

Supplement: Supplementary file 2 — Suppl. Fig. 1 [file 41388_2020_1273_MOESM2_ESM.tif]

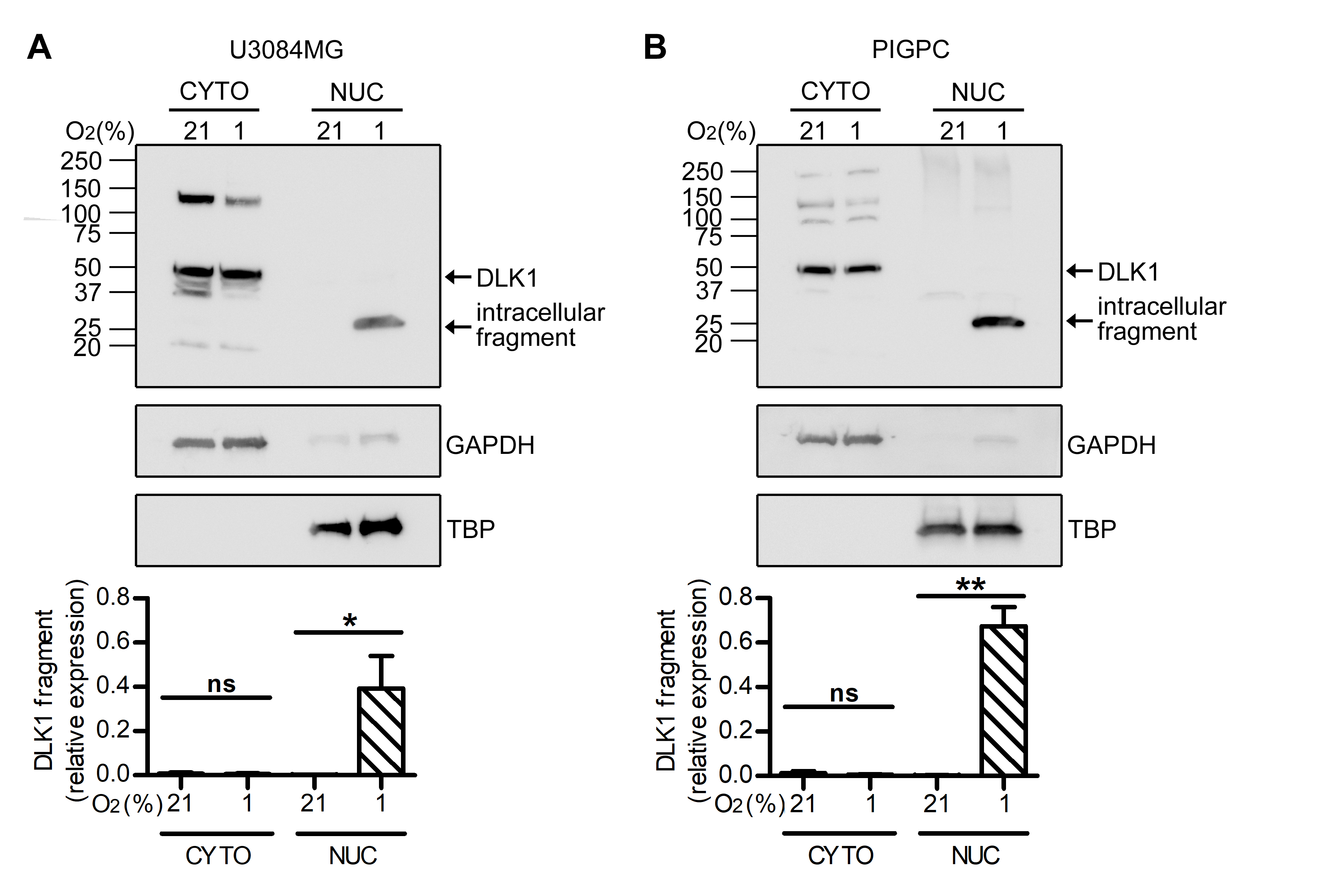

Supplement: Supplementary file 3 — Suppl. Fig. 2 [file 41388_2020_1273_MOESM3_ESM.tif]

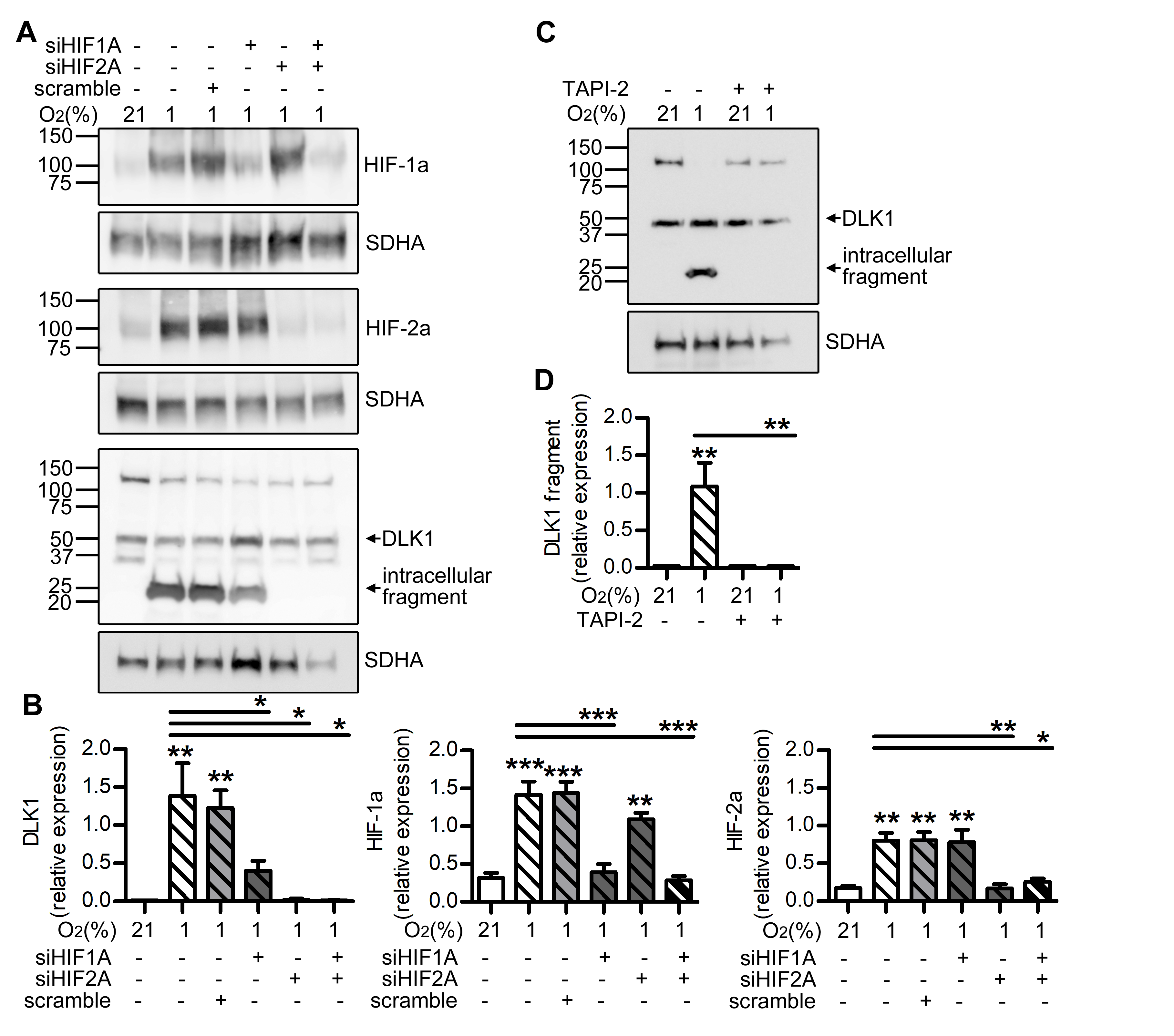

Supplement: Supplementary file 4 — Suppl. Fig. 3 [file 41388_2020_1273_MOESM4_ESM.tif]

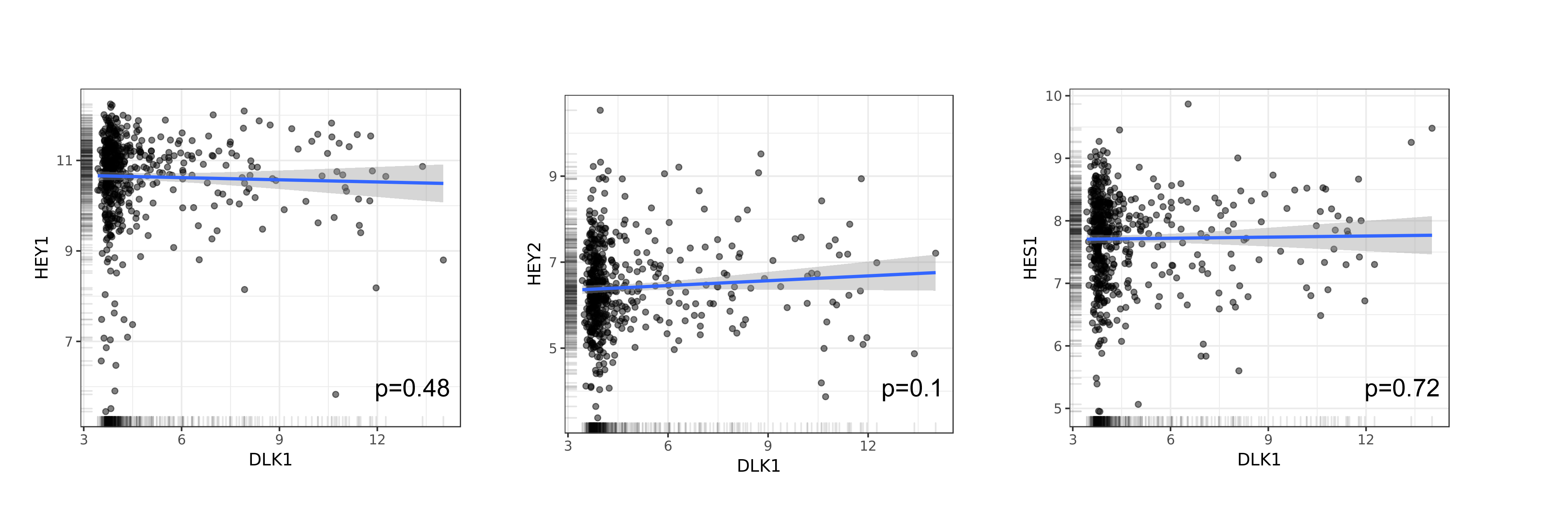

Supplement: Supplementary file 5 — Suppl. Fig. 4 [file 41388_2020_1273_MOESM5_ESM.tif]

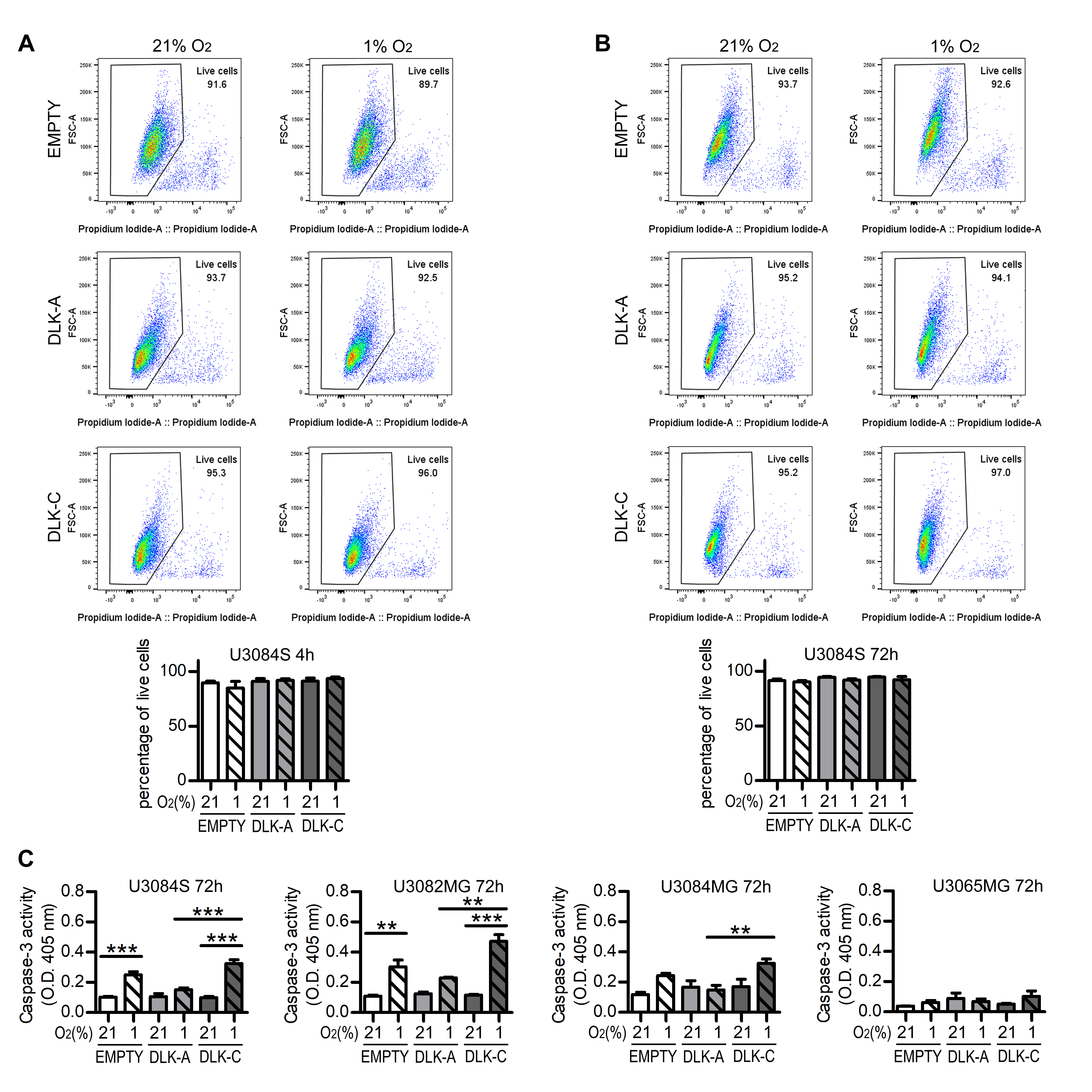

Supplement: Supplementary file 6 — Suppl. Fig. 5 [file 41388_2020_1273_MOESM6_ESM.tif]

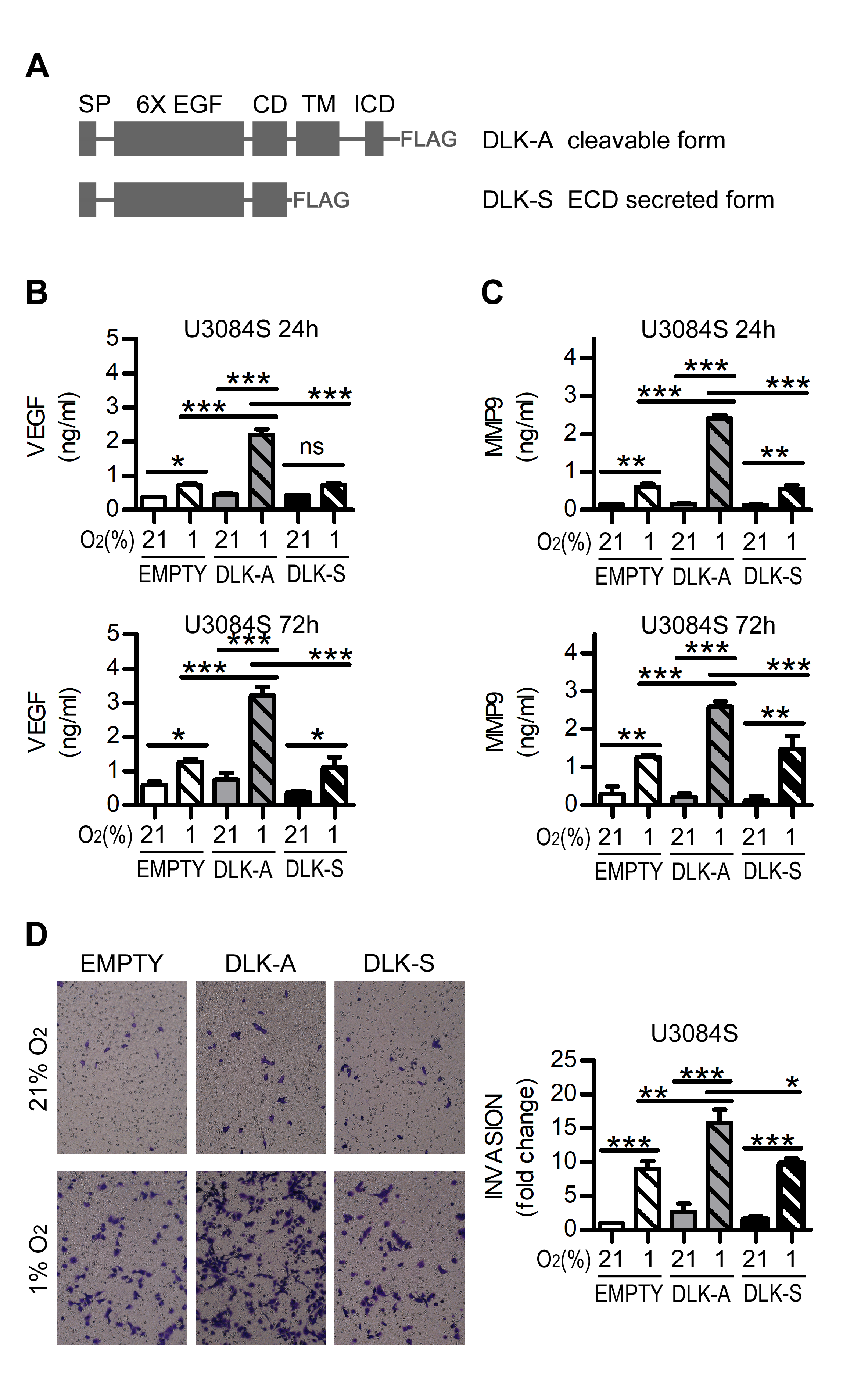

Supplement: Supplementary file 7 — Suppl. Fig. 6 [file 41388_2020_1273_MOESM7_ESM.tif]
