# Supplementary material for: Hypoxia-induced release, nuclear translocation, and signaling activity of a DLK1 intracellular fragment in glioma
Source: Oncogene. 2020 Mar 24;39(20):4028–44. doi: 10.1038/s41388-020-1273-9 (PMC7220882; doi:10.1038/s41388-020-1273-9)
Supplement: Supplementary file 8 — Suppl. Table 1 [file 41388_2020_1273_MOESM8_ESM.docx]

| **Kinase** | **DLK A (mean±sd)** | **DLK C (mean±sd)** |
| --- | --- | --- |
| p53 S46 | 627058,375±99324,03 | 2183281,75±420691,55 *** |
| p53 S392 | 2407696,25±495485,54 | 4692844,00±385100,08 *** |
| CREB S133 | 1639555,5±11381,63 | 2059277,25±88935,19 *** |
| p70 S6 Kinase T389 | 141104,1±11785,61 | 202598,88±9427,16 *** |
| p70 S6 Kinase T421/S424 | 175025,5±13232,44 | 238466,25±4404,46 *** |
| PLC-g1 Y783 | 187896,55±5193,53 | 228125,43±7146,53 *** |
| FAK Y397 | 1397486,5±46213,68 | 1891274,25±115692,36 *** |
| JNK1/2/3 T183/Y185, T221/Y223 | 2948774,5±37185,62 | 3277189,00±138220,38 ** |
| p53 S15 | 432043,675±84310,56 | 1154720,55±378291,85 ** |
| Akt 1/2/3 T308 | 221970,7±41769,40 | 342601,85±42038,31 ** |
| TOR S2448 | 703614,175±24827,30 | 834122,78±62631,73 ** |
| PRAS40 T246 | 3955570,25±291759,40 | 4941248,50±159321,90 ** |
| c-Jun S63 | 534019,925±93494,76 | 726624,40±64038,05 * |
| Lck Y394 | 428707,15±28183,35 | 526335,28±44059,64 ** |
| Yes Y426 | 927903,75±54363,60 | 1029770,83±38154,80 * |
| Fgr Y412 | 306639,575±23923,90 | 392502,65±45343,99 * |
| p38a T180/Y182 | 1351149,25±44824,61 | 1427858,75±142095,30 |
| ERK1/2 T202/Y204, T185/Y187 | 2983248,5±216321,64 | 2941589,25±284615,54 |
| GSK-2a/b S21/29 | 6359344,75±433690,38 | 6291274,75±326088,79 |
| EGFR Y1086 | 595912,9±25285,32 | 685983,73±85958,26 |
| MSK1/2 S376/S360 | 1360525±109519,68 | 1424374,25±159665,47 |
| AMPKa1 T183 | 911210,825±53180,26 | 885043,10±65396,61 |
| Akt 1/2/3 S473 | 4799891,75±879506,61 | 4862085,50±364439,94 |
| HSP27 S78/S82 | 635740,425±60038,11 | 642334,55±29648,70 |
| AMPKa2 T172 | 868975,175±19066,47 | 826405,68±33702,55 |
| b-CATENIN | 1507159,00±94342,01 | 1719787,75±144696,53 |
| Src Y419 | 820440,65±45927,09 | 956371,83±120665,99 |
| Lyn Y397 | 651049,25±60102,65 | 816831,15±137988,89 |
| STAT2 Y689 | 1001781,92±4757,23 | 1041949,05±62560,04 |
| STAT5a Y694 | 402290,57±53340,68 | 492190,20±81040,94 |
| Fyn Y420 | 611982,00±32963,77 | 696344,23±100258,51 |
| STAT6 Y641 | 886031,92±128098,75 | 913294,73±61045,69 |
| STAT5b Y699 | 445283,85±51157,87 | 448847,58±37678,45 |
| Hck Y411 | 659774,93±43891,28 | 817483,48±122269,10 |
| Chk-2 T68 | 1312364,75±147891,48 | 1288721,50±98103,94 |
| PDGF Rb Y751 | 409178,60±30870,69 | 443339,73±27601,00 |
| STAT5a/b Y694/Y699 | 795682,30±85055,36 | 794645,08±74650,19 |
| RSK1/2/3 S380/S386/S377 | 170400,33±50339,89 | 229322,08±19353,66 |
| eNOS S1177 | 125536,35±41307,32 | 135840,65±17108,46 |
| STAT3 Y705 | 216306,78±24499,20 | 235045,40±29140,79 |
| p27 T198 | 166603,03±36408,15 | 154552,58±23495,49 |
| STAT3 S727 | 305365,38±86474,42 | 370974,65±59981,44 |
| WNK1 T60 | 2140537,25±69086,99 | 2130110,75±141914,98 |
| PYK2 Y402 | 228209,53±17803,67 | 240820,25±12300,79 |
| HSP60 | 4020061,75±221256,35 | 3826283,50±273596,49 |
