# Supplementary material for: Hypoxia-induced release, nuclear translocation, and signaling activity of a DLK1 intracellular fragment in glioma
Source: Oncogene. 2020 Mar 24;39(20):4028–44. doi: 10.1038/s41388-020-1273-9 (PMC7220882; doi:10.1038/s41388-020-1273-9)
Supplement: Supplementary file 9 — Suppl. Table 2 [file 41388_2020_1273_MOESM9_ESM.docx]

|  | DLK A |  | DLK C |  |  |
| --- | --- | --- | --- | --- | --- |
|  | mean | sd | mean | sd |  |
| VEGF | 1570059,00±  211604,59 | | 523747,98± | 64169,14 | *** |
| MMP-9 | 161866,78±  24056,29 | | 67968,39± | 3083,89 | *** |
| angiopoietin-like 4 | 334173,40± | 19600,53 | 153067,95± | 33719,78 | *** |
| p53 | 2309075,00± | 133179,11 | 3677530,50± | 297905,55 | *** |
| p27/kip1 | 1050077,33± | 57127,48 | 593261,78± | 94651,58 | *** |
| Cga/b (HCG) | 1067406,75± | 58164,92 | 713947,55± | 16308,93 | *** |
| IL-18 Bpa | 367197,90± | 37945,17 | 192115,08± | 19824,12 | *** |
| CCL2/MCP-1 | 106039,19± | 28446,03 | 283816,33± | 27355,26 | *** |
| FoxC2 | 364256,85± | 51838,89 | 201251,80± | 12208,61 | *** |
| VCAM-1/CD106 | 556740,08± | 16463,26 | 799092,73± | 79258,63 | *** |
| kallikrein 3/ PSA | 652250,18± | 113395,14 | 327717,33± | 22534,77 | ** |
| Dkk-1 | 1057685,25± | 45236,55 | 1369125,75± | 115794,56 | ** |
| Era/NR3A1 | 402232,48± | 22894,98 | 265574,60± | 49268,04 | ** |
| ErbB2 | 523890,83± | 25389,77 | 656181,28± | 59274,00 | ** |
| Ho-1/HMOX1 | 485047,43± | 40726,37 | 659579,23± | 48558,47 | ** |
| Progranulin | 953490,18± | 17947,41 | 1222250,00± | 122205,76 | ** |
| Survivin | 1022199,65± | 187432,54 | 1751604,75± | 325402,66 | ** |
| HIF-1a | 1736754,50± | 142717,17 | 2180938,75± | 45303,09 | ** |
| u-Plasminogen Activator/Urokinase | 634229,98± | 15284,10 | 737691,85± | 46895,36 | ** |
| E-Cadherin | 157093,33± | 8502,53 | 120136,19± | 20258,31 | * |
| Cathepsin D | 1447514,75± | 52566,01 | 1107391,85± | 202820,04 | * |
| IL-6 | 442656,73± | 27632,61 | 375336,48± | 36533,58 | * |
| CXCL8/IL-8 | 1111339,50± | 97464,23 | 852752,45± | 142586,27 | * |
| Kallikrein 5 | 179123,50± | 27072,19 | 128077,73± | 9772,74 | * |
| CCL-3/MIP-1a | 240677,75± | 17803,70 | 183680,10± | 30287,68 | * |
| MMP-3 | 467145,00± | 86919,07 | 332110,83± | 44045,73 | * |
| Vimentin | 2374325,00± | 89951,08 | 2038484,00± | 175086,40 | * |
| a-fetoprotein | 384616,90± | 37234,25 | 377768,40± | 52076,11 |  |
| amphiregulin | 252533,75± | 84980,62 | 255458,98± | 83299,06 |  |
| angiopoietin-1 | 245332,58± | 95079,12 | 248608,53± | 57479,59 |  |
| ENPP-2/Autotaxin | 208139,65± | 14177,77 | 190258,13± | 35950,91 |  |
| Axl | 571612,18± | 24028,16 | 515314,00± | 65768,67 |  |
| BCL-x | 235989,00± | 18002,28 | 257680,30± | 21465,80 |  |
| CA125/MUC16 | 223171,83± | 24725,54 | 190160,83± | 78278,39 |  |
| VE-cadherin | 485124,85± | 17960,23 | 396183,75± | 74412,93 |  |
| CapG | 1959317,75± | 275094,85 | 1797883,25± | 137075,90 |  |
| carbonic Anhydrase IX | 2191077,50± | 147783,53 | 2151866,00± | 342746,75 |  |
| Cathepsin B | 1043823,43± | 83397,72 | 1023223,55± | 238452,38 |  |
| cathepsin S | 526874,83± | 19435,63 | 568683,90± | 81469,02 |  |
| CEACAM-5 | 167623,60± | 9135,51 | 161257,40± | 11925,25 |  |
| Decorin | 397860,53± | 41629,38 | 359396,75± | 56513,91 |  |
| DLL1 | 447453,65± | 24408,99 | 380903,28± | 97329,91 |  |
| EGF R/ErbB1 | 3276966,00± | 127776,56 | 3044848,00± | 366740,98 |  |
| Endoglin/CD105 | 383974,10± | 66834,03 | 334555,68± | 74577,27 |  |
| Endostatin | 544797,40± | 26454,66 | 591302,90± | 178484,51 |  |
| Enolase 2 | 4404344,00± | 125732,65 | 4033208,00± | 560888,30 |  |
| eNOS | 253220,58± | 14388,74 | 200381,10± | 42485,10 |  |
| EpCAM/TROP1 | 187930,00± | 10077,54 | 167190,05± | 24472,39 |  |
| ErbB3/Her3 | 188025,90± | 31472,10 | 189587,30± | 39264,90 |  |
| ErbB4 | 2064177,00± | 88756,61 | 1772045,00± | 352092,48 |  |
| FGF basic | 2185049,00± | 167586,42 | 2010908,75± | 188777,85 |  |
| Fox01/FKHR | 1585093,75± | 602384,49 | 1184523,50± | 32856,70 |  |
| galectin-3 | 3406782,00± | 214267,23 | 3166390,00± | 280550,58 |  |
| GM-CSF | 313430,08± | 42144,77 | 259466,00± | 20828,25 |  |
| HGF R/c-Met | 476177,70± | 25514,50 | 462032,20± | 10683,85 |  |
| HNF-3b | 221277,68± | 13742,08 | 198559,65± | 11037,85 |  |
| ICAM-1/CD54 | 176153,83± | 26929,74 | 153304,28± | 11714,77 |  |
| IL-2Ra | 250322,58± | 37000,35 | 222871,83± | 29311,79 |  |
| kallikrein 6 | 186904,00± | 38853,87 | 132822,70± | 24766,45 |  |
| Leptin | 182505,83± | 27174,33 | 157836,60± | 18228,24 |  |
| Lumican | 174603,68± | 31635,13 | 147715,80± | 25882,18 |  |
| CCL-8/MCP-2 | 271726,43± | 59529,65 | 209655,80± | 26737,35 |  |
| CCL-7/MCP-3 | 163357,58± | 39217,48 | 117482,80± | 12419,76 |  |
| M-CSF | 1844339,50± | 100015,95 | 1792234,50± | 102469,31 |  |
| Mesothelin | 568665,28± | 63311,93 | 501723,18± | 44041,09 |  |
| CCL-20/Mip-3a | 319923,65± | 66530,29 | 225607,53± | 48104,19 |  |
| MMP-2 | 717146,65± | 26015,58 | 677182,43± | 80684,35 |  |
| MSP/MST1 | 335779,58± | 124056,23 | 217007,25± | 27160,04 |  |
| MUC-1 | 183637,93± | 34166,15 | 170742,28± | 11710,28 |  |
| nectin-4 | 161622,00± | 35719,90 | 152919,90± | 4775,36 |  |
| Osteopontin (OPN) | 2472246,00± | 199771,30 | 2589062,25± | 419139,61 |  |
| PDGF-AA | 288005,00± | 108003,49 | 255471,58± | 52025,66 |  |
| CD31/PECAM-1 | 176168,33± | 60117,89 | 140633,78± | 22669,98 |  |
| progesterone R/NR3C3 | 225905,68± | 24682,08 | 195163,60± | 18587,90 |  |
| Prolactin | 284049,65± | 42580,53 | 244269,63± | 53066,89 |  |
| Prostasin/Prss8 | 167521,68± | 33478,14 | 141232,95± | 15484,23 |  |
| E-selectin/CD62E | 127943,33± | 41283,45 | 117834,34± | 16691,95 |  |
| Serpin B5/Maspin | 108053,67± | 26900,25 | 97338,80± | 8269,87 |  |
| Serpin E1/PAI-1 | 972485,90± | 57640,93 | 845467,50± | 107710,66 |  |
| Snail | 419606,35± | 37077,76 | 486049,45± | 169595,32 |  |
| SPARC | 1538274,50± | 31469,09 | 1511935,75± | 288526,49 |  |
| tenascin C | 1549639,75± | 60719,03 | 1827154,25± | 223521,02 |  |
| Thrombospondin-1 | 259702,25± | 64955,64 | 239146,25± | 6210,51 |  |
| Tie-2 | 214918,08± | 25580,39 | 223717,98± | 26103,01 |  |
